# Cancer and Thrombotic Risk: The Platelet Paradigm

**DOI:** 10.3389/fcvm.2017.00067

**Published:** 2017-11-07

**Authors:** Elizabeth C. Lee, Scott J. Cameron

**Affiliations:** ^1^Aab Cardiovascular Research Institute, Rochester, NY, United States; ^2^Department of Medicine, Division of Cardiology, University of Rochester School of Medicine, Rochester, NY, United States; ^3^Department of Surgery, Cardiac Surgery, University of Rochester, Rochester, NY, United States

**Keywords:** thrombosis, platelet activation, platelet dysfunction, cancer progression, dysregulated platelets, antiocoagulation

## Abstract

Hematologic malignancies and solid tumors increase the risk of venous and arterial thrombosis and contribute greatly to patient morbidity and mortality. Thrombosis occurs when the intricate balance of circulating antithrombotic and prothrombotic blood elements are disrupted. In recent years, the interplay between paraneoplastic cells and platelets has become apparent, with a change in platelet phenotype causing dysregulated platelet activity. This review discusses mechanism of thrombosis in cancer, evidence for using drug therapy, and exciting research efforts to understand and hopefully control aberrant thrombotic events in patients with cancer.

## Introduction

Patients afflicted with malignancies have a known propensity for thrombosis, with twenty to thirty percent of all first venous thromboembolisms (VTEs) associated with cancer (1). This thrombophilia may be a consequence of alterations in coagulation factor quantity and activity due to the underlying disease itself, the treatment for the disease, and alterations in platelet function (2–4). In addition, new evidence suggests that interactions between platelets and malignant cells lead to platelet activation and increased incidence of thrombosis (5, 6). The specific malignancy plays a role in the propensity for thrombosis, with higher rates associated with solid tumors of the pancreas, ovary, and brain in addition to hematologic malignancies, particularly, Hodgkin lymphoma (7, 8). While VTE is a well-documented risk in patient with malignancies, thrombosis in the arterial tree such as that observed in acute coronary syndrome (ACS) is also more common in patients with malignancy (9). Through understanding the pathophysiology of thrombosis in malignancies at the cellular level, we can investigate therapies and postulate future research avenues.

## The Role of Platelets in Cancer

There is an emerging body of evidence addressing the interactions between platelets and cancers. Not only do cells of paraneoplastic origin activate platelets, but platelets themselves appear to play a role in cancer propagation and metastatic spread in a process sometimes described as “tumor education.” Direct interaction with tumor cells induces platelet aggregation in experimental pancreatic, colorectal, and renal cell lines (6). Additionally, cancer cells directly excrete thrombin and other mediators, which interact with platelet surface receptors *via* PAR-1 and PAR-4 receptors (thrombin is the agonist), P2Y_12_ receptor (ADP is the agonist), and the thromboxane receptor (thromboxane A2 is the agonist). Tumors also secrete matrix metalloproteinases (MMPs) and IL-6, which have been shown to activate platelets directly (6, 10, 11). Specific study of human small cell and non-small cell lung cancer cell lines revealed *in vitro* induction of platelet aggregation both through direct cellular interactions observed under electron microscopy in SCLC and indirect cellular interactions *via* secreted thrombin and ADP mediators in NSCLC (12). Similarly, in a mouse xenograph of four different human pancreatic cell lines, two of the lines were found to express TF and release TF-positive microparticles—both known thrombogenic entities (5). As may be expected, only the TF-positive cell lines activated coagulation pathways in mice. In breast cancer cell lines, secreted MMPs led to platelet activation *via* cellular actions, which were independent of TF concentration: the platelets changed shape to form pseudopodia and demonstrated an increased concentration of activated GPIIb/IIIa surface receptors, which are then able to bind with fibrinogen and form stable platelet aggregates (13). Von-Willebrand Factor (VWF) also plays a role in platelet aggregation and recruitment of platelets to the vascular endothelium as demonstrated in patients with melanoma and mouse models of melanoma. This phenomenon may be related to tumor-derived vascular endothelial growth factor (VEGF) secretion, which mediates endothelial cell activation and, therefore, promotes VWF expression in the tumor vessel lumen—a biological process, which promotes platelet recruitment and atheroembolism (14).

The important interactions between platelets and malignant cells are increasingly clear as there is a growing body of evidence for the platelet’s role in metastatic spread of a variety of tumors. Early studies of renal sarcoma noted a correlation between the tumor’s ability to enhance platelet aggregation and the tumor’s metastatic potential (15). Platelet count itself also changes a patient’s metastatic risk. Patients with renal cell carcinoma and thrombocytosis had worse prognosis than those with normal platelet counts, again suggesting that platelets play a role in disease progression (16). Further investigation with *in vitro* and *in vivo* models has elucidated an intricate interplay between malignant cells and platelets, which propagates metastatic spread. When a tumor cell infiltrates the vasculature, it activates platelets and induces platelet aggregation around the tumor cell. This shields the tumor cell from the host immune system, allowing the tumor to evade the immune system and promoting survival. Additionally, the platelet releases microparticles, which promote blood vessel permeability and extravasation, allowing transport of the tumor cell to a new location. Finally, VEGF released by platelets promotes angiogensis both locally within the tumor and systemically throughout the vasculature (17, 18). Tumor angiogenesis promotes tumor growth (19). There may be additional mechanisms of tumor-platelet interactions yet elucidated, as recent study of a mouse melanoma model showed that platelets inhibit T-cell function, allowing the tumor to evade the immune system and metastasize (20). In contrast, at the bone marrow level, there is evidence that megakaryocytes are protective against bone metastasis of prostate cancer and breast cancer cells (21, 22). Additional study is needed on the specific interactions between platelet progenitors and cancer.

The role of platelets in metastatic spread leads to the hypothesis that antiplatelet agents will decrease tumor progression (23, 24). Although there is no guideline or recommendation for antiplatelet agents as a treatment of known malignancy, there is evidence that ticagrelor, a P2Y_12_ inhibitor, reduces metastases in murine models of melanoma and breast cancer (25). Rothwell et al. pooled a group of patients enrolled in randomized controlled trials of aspirin in vascular disease and performed a secondary analysis examining the incidence of distant metastases in patients who developed cancer both on and off aspirin (26). There was a significant decrease in distant metastases and death in patients taking aspirin who developed adenocarcinomas as opposed to those who were not taking aspirin. The use of aspirin did not change the risk of other fatal cancers (26). However, the role of antiplatelet agents in slowing malignant progression remains unclear. In a large population-based cohort study of patients who began low-dose aspirin therapy after diagnosis with colorectal cancer, there was no association with a reduction in colorectal cancer specific mortality (27). A meta-analysis of multiple cohort and one case-control study reached a similar conclusion that aspirin use after colorectal cancer diagnosis did not improve patient survival (28). In prostate cancer, aspirin use after diagnosis may only improve prostate cancer mortality in patients with high-risk cancer (29, 30). Overall, the evidence is not robust enough to advise routine use of antiplatelet agents as a component of the treatment armamentarium for cancer, though the use of antiplatelet agents in a personalized manner in select cases of cancer treatment should be investigated.

There are more robust data to support the prophylactic effect of aspirin in colorectal cancer. In fact, the language of the USPSTF recommendation for aspirin use combines primary prevention of coronary artery disease and colorectal cancer into the same statement and grade (31). Additionally, the use of aspirin in cancer prevention may extend to other solid tumors. An analysis of data from the Nurses’ Health Study and the Health Professionals Follow-up Study revealed a reduced incidence of overall cancer in subjects regularly taking aspirin (32). The largest effect was seen in gastrointestinal tract cancers, particularly colorectal cancer. There is evidence that low-dose aspirin reduces the risk of developing epithelial ovarian cancer; however, prior aspirin may not improve survival in patients once they receive a diagnosis of ovarian cancer (33, 34). Overall, additional studies are needed to better elucidate which specific malignancies may respond to aspirin either as a prophylactic measure or as part of a post-diagnosis therapy regimen.

## Cancer and Cardiovascular Risk

The relationship between malignancy and platelet activation may be an underlying mechanism for increased thrombotic events seen in patients with cancer. Additionally, platelets are significantly involved in the pathophysiology of ACS and thus the hypothesis that patients with cancer are at higher risk for coronary events is compelling. There are data that patients with occult cancer have a higher risk of coronary events even 2 years prior to cancer diagnosis, compared to control patients. This risk was found to be highest in patients with colorectal cancer (9). After cancer diagnosis, the risk of subsequent coronary disease events was highest in the first 6 months but persisted to 10 years after initial diagnosis. This risk was further increased by the presence of metastases (35). Additionally, there is a significantly increased risk of bare metal coronary stent thrombosis in patients with solid tumors (36). It is unclear how to further treat these patients beyond recommended dual-antiplatelet therapy since, at this time, evidence for using Factor Xa inhibitors of vitamin K antagonists is lacking and conceivably would increase the risk of bleeding.

Cancer treatment regimens themselves may also increase the risk of cardiovascular events. In a meta-analysis of seven randomized controlled trials examining the risk of thrombotic and cardiovascular events in women with breast cancer treated with tamoxifen or aromatase inhibitors, Cuppone et al. reported a slight increase in the pooled outcome of cardiovascular adverse events (37). The authors postulate this difference is related to the cholesterol raising effect of aromatase inhibitors. However, the specific risk of myocardial infarction related to aromatase inhibitor or tamoxifen therapy is unclear (38). Mediastinal therapeutic radiation is also an important risk factor for future cardiovascular disease. The European Society of Cardiology recommends preventative therapy with an antiplatelet agent as well as regular screening for cardiac disease beginning 10–15 years after initial radiation treatment (39).

The question remains of how to manage patients with cancer and myocardial infarction. In the short term, practitioners may be reticent to treat patients with ACS with antiplatelet agents, particularly, if the patient has thrombocytopenia as a pathophysiological consequence of malignancy or secondary to chemotherapy agents. However, aspirin has been shown to decrease all-cause mortality in patients with an acute myocardial infarction, even those with severe thrombocytopenia (40). This observation raises the concern that a low platelet count in the absence of bleeding should not be a reason to discontinue antiplatelet therapy in a patient with active coronary disease since the platelet population may be “dysregulated” with enhanced thrombotic potential. An additional study showed that both beta blockers and aspirin improve mortality in patients with known cancer and an acute myocardial infarction, whereas revascularization did not have a significant impact on mortality in this patient cohort (41). Thus, it remains important to adhere to guideline-directed medical therapies of ACS even in patients with malignancy.

## Cancer and Thromboembolism

The association between cancer and VTE is well-established and VTE is a dangerous complication of malignancy and chemotherapy treatments. Cancer is a hypercoagulable condition due to disease-related aberrations in the coagulation cascade. As discussed above, both platelets and tumor cells release tissue factor, which is one of the major factors in the extrinsic coagulation pathway. TF joins with factor VIIa to form a complex, which activates both factor IX to IXa and X to Xa, leading to thrombin formation and clots (42). Additionally, cancer procoagulant is a protease, which activates factor X independently of TF and has been detected in malignancies such as ovarian, colon, kidney, breast, prostate, and small cell lung cancer (43). Third, heparanase is an enzyme found in platelets, which enhances TF activity and is upregulated in some cancers. This leads to a positive feedback cycle of increased TF activation, which in turn activates platelets and promotes the release of additional heparanase (44). It is through these mechanisms that malignancies effect the coagulation cascade and promote thrombosis.

Additionally, the endothelium itself, endothelial modulators such as VEGF, and circulating blood cells play an important role in the thrombotic process. First, as a solid tumor grows, the hypoxic microenvironment of the tumor promotes reactive oxygen species generation by the mitochondria leading to release of angiogenic proteins such as VEGF and induction of transcription factors as well as increased endothelial permeability due to endothelial intracellular actin remodeling. The increased endothelial permeability exposes native and tumor extravascular TF expressing cells to circulating factor VIIa, initiating the coagulation cascade (45). Additionally, endothelial permeability allows for tumor cell metastasis, increased exposure of blood products to cancer-produced thrombin, and adhesion of pro-inflammatory cells to the vascular endothelium (45, 46). The malignancy’s direct action on platelet activation and aggregation then couples with the increase in pro-inflammatory leukocytes in the endothelium (5, 13, 47). Activated platelets and leukocytes interact to form microthrombi within the vasculature and these microthrombi adhere to the endothelium creating a nidus for larger thrombi to form (6). These processes of localized thrombosis are hypothesized as the underlying etiology of the histologic findings of vascular pseudopalisades around a necrotic core in glioblastoma multiforme (48). An area of additional interest regarding the role of platelets in thrombosis could examine abnormal megakaryoblasts in acute megakaryocytic leukemia, a rare AML subtype in adults (1% of AML cases), which can present with thrombocytopenia but has limited data on prognosis and treatment (49). Mechanisms for enhanced thrombosis in cancer are summarized in Table 1.

**Table 1:** Summary of pro-thrombotic elements observed in cancer.

Mediator	Origin	Mechanism
Tissue factor (TF)	○ Platelets ○ Malignant cell expressed ○ Malignant cell secreted microparticles	○ Activates platelets ○ Activates extrinsic clotting cascade ○ Binds with Factor Vll/Vlla and this complex activates Factor IX to IXa and Factor X to Xa ○ Ultimately increases activation of prothrombin to thrombin
Matrix metaloprotease	○ Native cell secreted ○ Malignant cell secreted	○ Activates platelets independent of TF ○ Increases platelet binding to fibrinogen increases platelet aggregation
Vascular endothelial growth factor (VEGF)	○ Native cell secreted ○ Malignant cell secreted	○ Increases VonWillebrand Factor expression on endothelial cells, promoting platelet aggregation ○ Increases endothelial permeability, exposing cells and clotting factors to extravascular TF ○ Draws pro-inflammatory leukocytes, which complex with platelets and form microthrombi
Cancer Procoagulant	○ Malignant cells only	○ Activates FX to FXa independent of TF
Heparanase	○ Platelets ○ Neutrophils ○ Monocytes ○ Malignant cell cytoplasm malignant cell secreted	○ Enhances TF activity ○ Activates VEGF, increases neoangiogenesis

The microscopic process of localized tumor thrombosis, neovascularization, and disruption of the coagulation cascade becomes important in clinical practice, as epidemiologic studies have found that 20–30% of all first VTE events are cancer-associated, with a cumulative incidence of VTE in cancer patients of 1–8% (1, 50). Diagnosis of thrombosis is a poor prognostic sign for patients with cancer. In a prospective study of patients receiving cancer chemotherapy, progression of disease was the leading cause of death, closely followed by thrombosis and infection (51). Thus, it is an issue of great importance with research focused both on the prevention of VTE and on appropriate treatment.

The risk of developing VTE depends on the time from initial cancer diagnosis, tumor origin, treatment modalities, and laboratory values. Pulmonary embolism (PE) may precede a diagnosis of lung cancer and also carries a sixfold higher risk than controls in the year following diagnosis (52). This risk is further elevated by receiving chemotherapy, with registry data indicating an eightfold increase in the risk of developing a PE compared to age-matched controls. Patients with pancreatic cancer and brain cancer have a higher risk of VTE and patients with prostate cancer and breast cancer have a lower risk. Additionally, surgical instrumentation and chemotherapy treatment increase a patient’s VTE risk (50). Further independent risk factors for VTE include high platelet count and higher leukocyte count (53).

Clinically, a patient’s VTE risk can be quantified using the Khorana Score. This is a comprehensive risk model validated in multiple cancer types, which uses parameters such as site of cancer, presence of anemia, platelet count, leukocyte count, and BMI to calculate an ambulatory patient’s risk of VTE and guide the decision for prophylaxis (54, 55). The model is recommended by the American Society of Clinical Oncology (56). Data suggest that inhibiting plasma Factor Xa with low molecular weight heparin is the prophylactic agent of choice to reduce occurrence of VTE; however, its use does not impact survival (50, 57). Because of the known role of platelets in thrombus formation for patients with cancer, it is reasonable to hypothesize that aspirin would have a protective effect on VTE risk. In patients without malignancy, aspirin is useful for both prophylaxis and prevention of VTE recurrence (58, 59). However, in patients with malignancy, there is a trend to suggest that aspirin is effective in VTE prophylaxis, but the evidence is not strong enough to support routine use (60). If a patient with cancer does develop VTE, the treatment of choice remains low molecular weight heparin rather than vitamin K antagonists based upon open-label randomized controlled trials and a meta-analysis showing a decrease in recurrent VTE in cancer patients treated with LMWH compared to warfarin (50, 61).

## Conclusion/Further Study

*In vivo* and *in vitro* studies in human and animal models have described the interactions between platelets and cancer cells. With this background descriptive knowledge, it is a compelling hypothesis that antiplatelet agents are useful in decreasing malignant transformation as well as the occurrence of thrombosis. There is evidence for aspirin as a cancer preventative agent, particularly in colon cancer as reflected in the USPSTF guidelines. Unfortunately, the data for antiplatelet agents as an adjunctive treatment to chemotherapy in order to decrease the spread of malignancy is not as strong. Similarly, the use of antiplatelet agents for prophylaxis of VTE and other thrombotic events such as myocardial infarction has not been widely adopted. However, available data suggest that patients with cancer who develop ACS should be treated with guideline-based secondary prevention therapies, including aspirin even in the setting of thrombocytopenia (40). Areas of potential research include how to treat patients with malignancy and in-stent thrombosis despite dual antiplatelet therapy. Perhaps these patients would benefit from low molecular weight heparin treatment, though this would have to be carefully weighed against the risk of bleeding. Ongoing areas of interest include prophylaxis, treatment, and secondary prevention of thrombosis and VTE in cancer patients. Additionally, while the Khorana Score focuses on the risk of VTE, it may be helpful to incorporate malignancy into other risk scores such as the CHAD2S-VASC score for atrial fibrillation and embolic stroke risk. These are potential avenues of collaboration between the cardiovascular and oncology societies to formalize recommendations for this unique population of patients.

## Author Contributions

Both authors contributed equally to this work.

## Conflict of Interest Statement

The authors declare that the research was conducted in the absence of any commercial or financial relationships that could be construed as a potential conflict of interest.
